# Hair me out: Highlighting systematic exclusion in psychophysiological methods and recommendations to increase inclusion

**DOI:** 10.3389/fnhum.2022.1058953

**Published:** 2022-12-08

**Authors:** Courtney C. Louis, Christopher T. Webster, Lilianne M. Gloe, Jason S. Moser

**Affiliations:** Department of Psychology, Michigan State University, East Lansing, MI, United States

**Keywords:** EEG, hair cortisol, anti-Black racism, inclusion, hair-bias

## Introduction

Within the neuroscience field, there have been efforts to address the ways systemic racism has permeated and negatively affected our research practice and body of knowledge (Abiodun, 2019; Choy et al., 2021; Carter et al., 2022; Webb et al., 2022). Neuroscience methods that require access to the hair and scalp systematically exclude groups of people, particularly Black communities, over and beyond the embedded exclusionary factors in the broader human research landscape (Gatzke-Kopp, 2016; Roberts et al., 2020; Fulvio et al., 2021; Taylor et al., 2021; Goldfarb and Brown, 2022). Indeed, recent papers have highlighted the shortcomings of current neuroscience methods (Choy et al., 2021; Parker and Ricard, 2022; Webb et al., 2022). Recently, Bradford et al. (2022) discussed underrepresentation in psychophysiological research samples and offered insightful recommendations for researchers to improve inclusion. We amplify and extend these valuable efforts, with a particular focus on methods that require access to participants' hair, such as electroencephalography (EEG) and hair sample collection. We briefly review factors that have led to the systematic exclusion of underrepresented groups in psychophysiological research and synthesize practical recommendations for researchers to increase inclusion moving forward.

To understand systematic exclusion in neuroscience methods, it is essential to name the legacy of anti-Black racism and its impact on research practices. Many early empirical pursuits often aimed to provide scientific justification for the exclusion and continued maltreatment of Black populations (Kuria, 2014). There are well-documented instances of unethical and harmful research conducted with Black populations (Washington, 2006). Additionally, there is continued mistrust in institutions given present-day experiences of racism and discrimination in medical and academic settings (for examples, see: Barber et al., 2020; Hassen et al., 2021). These historical and current experiences continue to influence neuroscience research. For instance, underrepresentation of Black, Indigenous, and people of color (BIPOC) researchers leads to a lack of diversity in research samples (Buchanan and Wiklund, 2020; Roberts et al., 2020). Many scholars have highlighted the tendency for psychological and neuroscientific research to primarily include western, educated, industrialized, rich, and democratic (WEIRD) samples (Henrich et al., 2010). Such research has also historically excluded BIPOC individuals and women (Taffe and Gilpin, 2021; Taylor et al., 2021), with individuals at the intersection of different marginalized identities (e.g., BIPOC women) being even less represented (Spates, 2012; Kuria, 2014). Exclusion in neuroscience research occurs despite evidence that suggests Black and POC participants are willing to participate in research overall (Wendler et al., 2005; Jones and Neblett, 2017; Manns-James and Neal-Barnett, 2019). This lack of representation has harmed our ability to make scientific progress, as findings commonly thought to be “generalizable” often only speak to a subset of WEIRD and White people and perpetuates harm onto BIPOC communities.

## EEG and hair sample collection: Highlighting exclusion within methods that require contact with hair

The methods employed in neuroscience research often serve as an indirect source of systematic exclusion, in that the methods themselves lead to consistent exclusion of specific populations. The source of this exclusion lies in the inadequacy of a given method to accommodate people with a variety of phenotypic traits, a direct form of “phenotypic bias” (Webb et al., 2022). When access to a participant's hair is required, even without the use of equipment, as is the case for hair sample collection, phenotypic bias can still be present and affect research practices methods and contribute to underrepresentation in research samples (Manns-James and Neal-Barnett, 2019; Choy et al., 2021). To highlight this, we focus our discussion on how EEG and hair sample collection to assay for cortisol results in the exclusion of Black participants, in particular.

## Lack of inclusive methodologies

Many EEG devices require access to the scalp to measure electrical brain activity. Thicker (i.e., coarser) and curlier hair can make access to the scalp more difficult when applying conductive electrode gel. Conductive gel acts as a bridge to establish the proper connection between the scalp and electrodes and can result in poor signal quality if access to the scalp is impeded. When EEG devices are used clinically, poor signal quality can affect clinical diagnosis and contribute to a burdensome experience for patients (Etienne et al., 2020). Researchers have attempted to compensate for the current limitations of EEG devices by applying more conductive gel to help establish a connection. However, this can result in the additional gel spreading across the scalp and bridging electrodes, which reduces spatial resolution (Etienne et al., 2020), and discomfort for the participant who is left with an abundance of hair gel to remove afterward. It is therefore common for EEG researchers to exclude participants with thick, curly hair due to poor data quality (Choy et al., 2021).

Most extant protocols for collecting hair samples to assay for cortisol do not account for differences in hair texture (e.g., curliness or thickness; Russell et al., 2012; Wright et al., 2018). Indeed, most require several centimeters of hair to be available for collection. Accounting for hair texture is necessary for determining the accurate length of hair samples and ensures that hair collection minimizes damage to the participant's hair (Wright et al., 2018). Using traditional protocols created for straight hair textures, hair cortisol researchers may exclude some individuals with curly hair because such individuals' hair may be considered too short (Wright et al., 2018).

In addition to methodologies being unaccommodating of thick and curly hair textures, these methodologies are also less well suited to hairstyles such as braids, twists, cornrows, or locs that are more likely to be worn by Black individuals. Individuals from various backgrounds may also wear extensions or wigs. Participants may have to partially or fully undo hairstyles for research studies, which may influence their participation. For instance, a recent study found that nearly half of Black women participants who declined to provide a hair sample reported doing so because they had hairstyles that would make accessing their natural hair more difficult (Manns-James and Neal-Barnett, 2019). Many of these hairstyles can take significant time to remove and can be quite expensive to redo, leading to increased cost and burden of participating in EEG and hair cortisol studies.

## Lack of inclusive staff training

Even if participants with thick, curly hair, or the aforementioned hairstyles are enrolled, research staff may not be trained or prepared to have respectful discussions with participants about their hair to facilitate data collection. Moreover, negative interactions with untrained staff can be harmful to research participants if disparaging or devaluing statements are made about their hair. For example, study staff may make statements about certain hair textures or styles being “bad,” “difficult,” or “undesirable” when difficulties in data collection arise. In addition, pervasive racial bias about hair textures and hairstyles may be communicated to participants during the data collection process (MacFarlane et al., 2017; Mbilishaka et al., 2020). Such interactions likely contribute to systematic disengagement of diverse populations from participating in EEG and cortisol studies. Finally, cultural and religious differences surrounding the value of hair can also influence participation in research that requires access to the hair/scalp. For example, individuals who wear headscarves may not feel comfortable removing their headscarves around male research staff or others for various reasons. This can be a barrier to participation if there are no female researchers on the research team. To our knowledge, there are no published recommendations for accommodating participants who wear headscarves in EEG or hair cortisol studies, and, therefore, such individuals may be less likely to participate. In addition, some individuals may not want to provide hair samples because their natural hair has cultural or religious significance (Manns-James and Neal-Barnett, 2019).

## Foundations of these methodological/training limitations

The limitations of both EEG and hair collection methods may have led to exclusionary practices in neuroscience research, such as biased exclusionary criteria, increased financial burden on BIPOC participants, and harmful interactions with study staff. The limitations of these methodologies and staff training are likely related to the lack of diversity among researchers who developed them. For instance, less than 5% of psychologists and neuroscientists identify as BIPOC researchers (Society for Neuroscience, 2017; Lin et al., 2018). Reviewing operating manuals for popular EEG devices (i.e., ActiveTwo, NeuroScan, Brain Products) revealed no explicit instructions for EEG setup on participants with thick, curly hair or any mention of different hair textures or styles. Visual depictions of EEG setup only included images of individuals with straight hair textures. The operating manuals from these popular EEG devices highlight the extent to which EEG device manufacturers have neglected individual differences in hair texture.

## Recommendations

We present recommendations based on extant research to increase inclusivity in neuroscience research using physiological methods that involve contact with hair in Table 1. First, we recommend that researchers increase collaboration with BIPOC researchers. Author positionality directly affects the ways in which research is conducted (Taylor and Rommelfanger, 2022). The general standard to uphold scientific objectivity may often blind researchers to the legacy and current effects of anti-Black racism, and how it continues to affect our research practices. Therefore, collaborating with BIPOC researchers allows for diversity in scientific thought and ultimately improves our research questions, research ethics, and development of novel methodological solutions. For instance, Etienne et al. (2020) have introduced SEVO (Haitian Kreyól for “brain”) electrodes that allow direct access to the scalp for individuals with thick and curly hair. SEVO electrodes leverage a conventional Black hairstyle (i.e., cornrows) to improve EEG application, and employ attachments designed similar to hair barrettes to secure electrode placement and reduce the signal-to-noise ratio. Etienne et al., (2020) modified EEG design provides an innovative solution that improves data quality and participants' experience by addressing the limitations of many EEG devices.

**Table 1:** Recommendations for researchers to improve inclusive practices in EEG and hair cortisol research.

**Recommended broad changes**	**Specific recommendations across methods**		
Collaborate with BIPOC researchers.	Review the extant literature for recommendations by BIPOC researchers to increase inclusion of research participants and cite such researchers (Cundiff, 2012; Roberts et al., 2020; Zurn et al., 2020; Buchanan et al., 2021; Smith et al., 2021; Bradford et al., 2022; Webb et al., 2022). Collaborate with BIPOC researchers at all levels (i.e., undergraduates, graduate students, staff, post-doctoral fellows, and junior and senior faculty). Think critically about how to incorporate race into neuroscience research (Carter et al., 2022; Kaiser Trujillo et al., 2022).		
	**Specific recommendations across methods**	**EEG-specific recommendations**	**Hair cortisol specific recommendations**
Train research staff to promote inclusion in discussing and working with participants with diverse hair textures and styles.	Researchers should be well-versed in the diversity of hairstyles, textures, and care. Staff training should include refraining from value-based language about hair (e.g., “good hair” vs. “bad hair”). Researchers should accommodate participants who may hold cultural values around who is allowed to see and access their hair to best accommodate their needs.	Richardson and colleagues provide inclusive guidelines for hair preparation for EEG data collection and a description of hair characteristics and care (https://hellobrainlab.com/research/eeg-hair-project/). Consider prioritizing electrode placement based on research aims (e.g., focus on frontal or parietal electrodes needed for specific ERPs) instead of the whole scalp. If challenges during data collection arise, staff should communicate the limitations of the equipment rather than make negative statements about hair.	
Accommodate participants with diverse hairstyles and textures.	Provide participants with a video or visual demonstration of the EEG or hair collection process to increase transparency and describe how the data will be used. Provide a private setting for participants to ask questions about EEG/hair collection procedures. Schedule EEG or hair collection visits between hair appointments. Consider employing a beautician well-versed in working with Black hair to redo hairstyles following EEG collection or taking hair samples for cortisol (Manns-James and Neal-Barnett, 2019).^*^ Consider increased compensation in cases where excess time is required for a participant to modify or undue hairstyles for participation (Manns-James and Neal-Barnett, 2019).^*^	Researchers should have an open dialogue with all participants about their hair including understanding the participant's comfort level after thoroughly explaining procedures. Purchase add-ons to EEG equipment (Krishnan et al., 2018; Etienne et al., 2020).^*^ Researchers should advocate for more inclusive technology for all hair textures and styles (Robinson et al., 2022).	Consider that teaching participants self-collection of samples may increase participant comfort and sense of respect for the cultural significance of their hair. Collect a hair sample at a hair salon during a participant's scheduled hair appointment to reduce participant burden.

* We recognize that these particular recommendations require more resources than are available to some research teams to implement immediately. Therefore, we encourage researchers to strive toward these recommendations whenever possible but encourage the use of the recommendations without asterisks when resources are limited.

Second, we encourage increased research training on hair types and styles. Understanding differences in hair types and styles is critical for preparing hair for EEG and hair sample collection, communicating steps to research participants, and promoting a more inclusive environment. We encourage researchers to go about this process with cultural humility (as opposed to cultural competence), which involves the dual praxis of self-reflection and continuous learning (Yeager and Bauer-Wu, 2013).

Third, we recommend that researchers strive to accommodate all hair textures and styles. Equipment and protocols must be altered to make them more accommodating of thick and curly hair. Others have suggested employing a beautician well-versed in working with Black hair to redo hairstyles following EEG collection or taking hair samples for cortisol (Wright et al., 2018). Researchers could also acknowledge the increased burden on BIPOC participants by offering additional compensation to those who need hairstyles to be removed and/or scheduling study visits before hair appointments (Manns-James and Neal-Barnett, 2019).

Additionally, researchers should collaborate with participants toward successful data collection, such as allowing participants to self-collect their own hair samples or working with participants to determine how to best collect data when access to their scalp is impeded. For example, researchers interested in fronto-central or centro-parietal neural signals (the most canonical locations for many common EEG/ERP metrics) could prioritize the placement of midline sites if access to other areas of the scalp is occluded. Finally, researchers should be conscious that certain cultural/religious practices dictate that only people of the same gender can see their hair. Therefore, we recommend conducting EEG and hair sample collection in a private space and that lab visits are adjusted, if needed, to meet the participant's needs.

## Conclusion

In sum, anti-Black racism continues to shape research practices that rely on physiological methods involving contact with hair. While it may not be explicit, the use of these methods has a significant impact on who participates in research studies. Blindly abiding by the limitations of equipment or protocols leads to underrepresented samples, limited science, and a body of knowledge that does not apply to many. We believe a critical starting point to move toward inclusion is to modify research lab practices. While we have specifically reviewed EEG and hair cortisol, it is vital for this critical reflection and action to take place across multiple phases of the research process for a variety of research methodologies. We hope these recommendations may provide practical steps for researchers to employ in their labs to improve inclusion and expand the applicability and relevance of neuroscience research beyond White and WEIRD individuals.

## Author contributions

CL, CW, and LG conceptualized and drafted the manuscript. All authors provided critical revision of the manuscript and approved the final version for submission.
